# Implications of Medical and Recreational Marijuana Laws for Neuroscience Research: a Review

**DOI:** 10.1007/s40473-020-00222-5

**Published:** 2020-10-20

**Authors:** Deborah S. Hasin, Efrat Aharonovich

**Affiliations:** 1 Department of Epidemiology, Mailman School of Public Health, Columbia University, 722 W 168th St, New York, NY 10032, USA; 2 New York State Psychiatric Institute, 1051 Riverside Dr, New York, NY 10032, USA; 3 Department of Psychiatry, Columbia University Medical Center, 1051 Riverside Dr, New York, NY 10032, USA

**Keywords:** Medical marijuana laws, Recreational marijuana laws, Cannabis use disorder, Neurocognitive impairments, Cannabis potency

## Abstract

**Purpose of Review:**

Review of US medical and recreational marijuana laws (MML and RML), their effects on cannabis potency, prevalence of non-medical cannabis use and cannabis use disorder (CUD) in adolescents and adults, and implications for neuroscience research, given what is known about the relationship of cannabis to neurocognitive impairments and underlying brain functioning.

**Recent Findings:**

Cannabis potency may be increasing faster in states with MML or RML than in other states. MML and RML have not impacted prevalence in adolescents but have consistently been shown to increase rates of adult non-medical use and CUD.

**Summary:**

Recent neurocognitive or neuroimaging studies may be more impacted by cannabis than studies conducted when MML and RML were less common. Neurocognitive or neuroimaging studies conducted in MML or RML states should carefully test potential participants for recent cannabis use. More research is needed on cannabis and cognition in medical marijuana patients.

## Introduction

Cannabis has been used in the United States since the 1800s, with public attitudes towards its acceptability and potential harmfulness varying considerably over time [1]. In 1970, only 12% of U.S. adults favored legalizing cannabis use [2], and the Federal Drug Enforcement Agency (DEA) defined cannabis as a Schedule 1 substance, i.e., no accepted medical use and high abuse potential [3]. Since then, legalization has steadily gained favor and many state marijuana laws have changed and become more permissive. Additionally, public perception of cannabis as a harmful substance has declined substantially among adolescents [4] and adults [5]. Consideration of the potential impact of these changes in laws and attitudes on behavioral neuroscience studies is warranted. In this review, we briefly consider the relationship of cannabis use to key areas of neurocognitive and brain functioning and describe medical and recreational cannabis laws. We then consider how changes in these laws have impacted cannabis potency, cannabis use patterns in adolescents and adults, and the increasing prevalence of people who use medical cannabis. In each of these areas, we consider the implications of the changes in the laws, and the resulting changes on cannabis characteristics and patterns for behavioral neuroscience studies.

Legalization of cannabis would not be relevant to considerations in behavioral neuroscience research if cannabis were unrelated to neurocognitive functioning. However, extensive research over the past 50 years, including hundreds of individual studies and many reviews and meta-analyses suggests that cannabis use is related to cognitive impairments and underlying brain functioning. Many debates remain in this area, including the causal nature of the relationships and reasons for some of the inconsistences in the literature. Nevertheless, broad agreement exists that acute, persistent, and heavy cannabis use affects cognition in many domains, e.g., memory, attention, and executive functioning.

Impaired attention is a key feature of acute cannabis intoxication [6•]. Whether such impairment persists following abstinence from cannabis is less clear. In the US adult general population, controlling for demographic characteristics, frequency of cannabis use in the past year predicted lower scores on an attention functioning self-report scale [7], while among those age 50 or older, people who were both current and former users (abstinent at least a year) had lower scores on attention than people who were never-users [8]. Many laboratory studies using objective attention measures have found post-abstinent deficits in attention that persist up to 30 days [6•, 9]. For example, comparing adolescents who were cannabis users and non-users [10, 11], deficits in attention were found in the adolescents who were users after 3–4 weeks of abstinence. Among participants age 16–26 with ADHD [12], cannabis use predicted attentional deficits 3 weeks post-abstinence, but ADHD symptoms did not. This study illustrates how cognitive impairment could be attributed to a psychiatric disorder if information on cannabis use was not also incorporated.

Memory is the cognitive domain most consistently reported as impaired by cannabis, with such effects found in adolescents, adults, and older adults [6•, 13•, 14–16]. Among adults, acute and chronic cannabis use has frequently been found to be associated with verbal and working memory impairments. These impairments are related to the duration, frequency, dose, and age of onset of cannabis use [15]. The most extensive evidence for impairment is within verbal learning and memory [14]. The effects of cannabis on working memory are less clear, perhaps because of the wide variety of working memory tasks that have been used to assess this domain [6•, 14, 15]. An open question in this area is the persistence of memory deficits post-abstinence. A recent review and meta-analysis found that overall, effects sizes for the relationship of cannabis use to cognitive deficits were diminished in studies with cognitive testing done after longer periods of abstinence in both adolescents and adults [13•]. However, this review did not address the issue of post-abstinence persistency by cognitive domain, leaving the issue unclear regarding memory. Earlier studies [17], including an earlier review [9], showed that chronic cannabis use was related to sustained post-abstinence deficits in memory function, so further research will be needed to better understand the persistence of memory deficits after cessation of cannabis use.

Executive functioning involves tasks of planning, reasoning, interference control, decision-making, and problem solving. In the US adult general population, frequency of cannabis use in the past year predicted lower scores on an executive functioning self-report scale [7], and among those age 50 and older, people who were former cannabis users (abstinent at least a year) had worse scores on executive functioning than people who were never-users [8]. Using neuropsychological test batteries in the laboratory to measure executive functioning, many although not all studies found relationships between cannabis use and impaired aspects of executive functions [6•, 13•, 18]. An example of one of these areas is inhibitory control/impulsivity, examined prospectively over several years [19], and through neuroimaging studies [20, 21]. However, due to heterogeneity of findings, reviews were inconsistent on whether cannabis use was more strongly related to impairments in executive functioning in adults in their mid-30s and older than in adolescents or young adults [6•] or among adolescents when compared with adults [22•].

A recent review of structural and functional neuroimaging studies compiled considerable information from positron emission tomography (PET) and magnetic resonance imaging (MRI) studies about brain structure and functioning related to cannabis use [23]. PET studies show that CB_1_ receptors are downregulated in people who were cannabis users, especially shortly after use. These changes are mainly located in the neocortex and limbic cortices, which regulate cognition, and in the ventral striatum, which is involved in reward and goal-directed behavior. Reduction of CB_1_ receptors in cannabis-dependent subjects returns to normal ~2 to 28 days after abstinence, although among those with chronic heavy use, the reduction in CB_1_ receptors may impact downstream systems that maintain changes underlying later behavioral characteristics of cannabis use disorder. Although evidence is somewhat conflicting, acute THC administration appears to cause increased dopamine release and neuronal activity, whereas long-term cannabis use is associated with blunting of the dopamine system [24], which is related to inattention. In cannabis-dependent individuals, PET studies of dopamine transporters (necessary for presynaptic dopamine reuptake) also show reduced availability in multiple brain areas, including the striatum. Glutamate also plays a role in mediating inhibitory control. Glutamatergic transmission is regulated through presynaptic terminal CB_1_ receptors that reduce glutamate release and are sensitive to THC. In the limited research in humans, chronic cannabis use appears to reduce levels of glutamate-derived metabolites in cortical and subcortical brain areas, while animal studies indicate that THC depresses glutamate synaptic transmission via CB_1_ receptor activation [25].

Human structural magnetic resonance imaging (MRI) studies show alterations in corticolimbic structures in those with CUD, e.g., prefrontal cortex (PFC), hippocampus and amygdala. The integrity of the orbitofrontal cortex (OFC within the PFC), which contributes to cognitive flexibility and decision-making, is often impaired in SUD and related to problem use. A similar sensitivity is evident in the hippocampus, a region central to learning and memory. An additional recent review and meta-analysis also showed smaller volume of the hippocampus and orbitofrontal cortex in people who were regular cannabis users compared with controls [26].

Functional magnetic resonance imaging (fMRI) studies of differences in brain functional alterations in people who were cannabis users and non-users while performing memory tasks showed that functional brain activation during the tasks was altered in the people who were cannabis users. The results suggested that the altered brain activation drove the deficits in memory performance [27]. More specifically, fMRI studies showed differential brain activity in people who were heavy cannabis users during neurocognitive tasks e.g., cost–benefit decision-making conditions, including reduced activity of the OFC and dorsolateral PFC but also increased cerebellar activity. Despite the well-documented negative cognitive impact of acute THC on working memory in drug-naïve individuals and people who were infrequent cannabis users, people who were experienced users often have normal working memory performance. However, neural networks associated with such cognitive function are not normal: people who were chronic heavy users had hyperactivation of frontal regions and networks underlying working memory. Collectively, these modifications suggest overcompensation of neural networks in people who were heavy users to achieve apparent normal executive function when cognitive demand is required.

## Medical Marijuana Laws

**Fig. 1:**
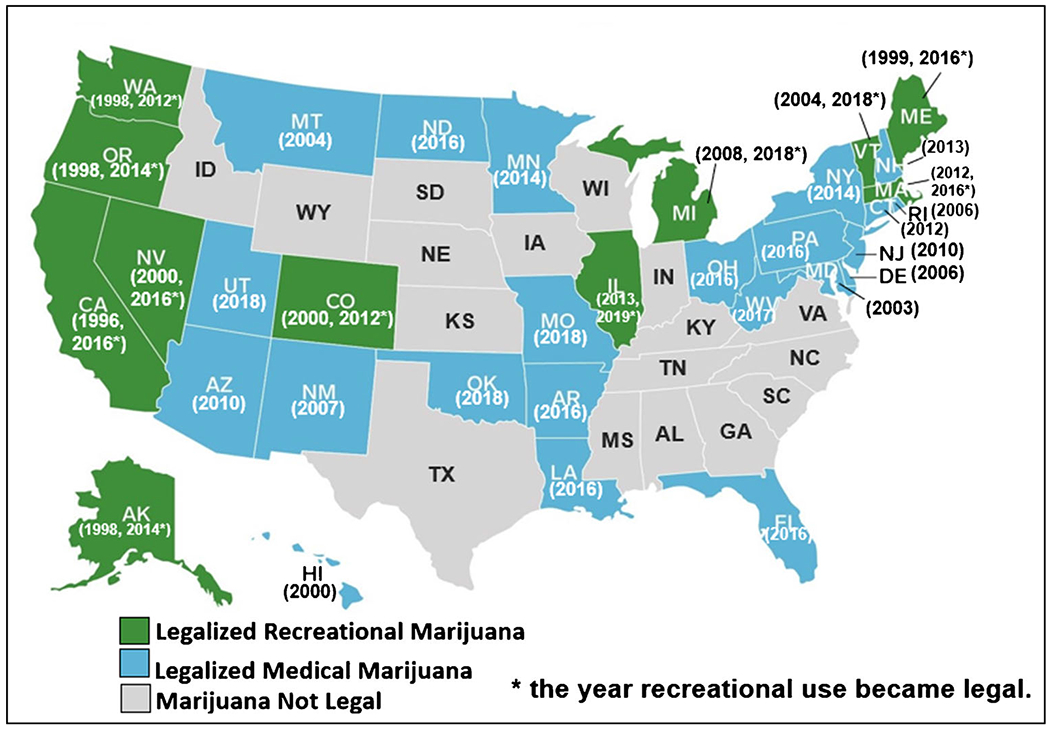
State Medical and Recreational Marijuana Laws, including years passed Abbreviations: MML – Medical Marijuana Law (adapted from Hasin et al., JAMA Psychiatry 2017).

In 1996, California became the first U.S. state to pass a medical marijuana law (MML) legalizing the use of cannabis for medical purposes. As of this writing, 34 states have MML, covering 67% of the US population (Fig. 1). State medical marijuana laws share the common feature that they permit legal use of cannabis to treat medical conditions if the person who used cannabis obtained medical authorization. However, the specific provisions of MML vary considerably [28] across states, and within states. For example, states can change the range and specificity of the permitted medical conditions and the permitted distribution outlets (e.g., dispensaries), permitted amounts per patient, etc. The restrictiveness or “medicalization” [29, 30] of MML varies as well. Concerns about MML have included their potential to increase problematic use of cannabis in the general population through several mechanisms, e.g., reducing perceived harmfulness, normalizing use [28, 31], and increasing availability via dispensaries and home cultivation [28, 31].

## Recreational Marijuana Laws

In 2012, Washington and Colorado became the first states to pass laws permitting legal use of marijuana for recreational purposes (RML). Eleven states now have such laws (all of which previously had MML), covering 28% of the US population, and several additional states are considering the passage of such laws (Fig. 1). Recreational marijuana laws (RML) permit legal sale and use of cannabis without the need for medical involvement. Potential benefits of such laws include reduction of discriminatory marijuana arrests of disadvantaged minorities [32, 33] and expansion of business opportunities, jobs, and tax revenues [34–36]. Cannabis is now a multi-billion-dollar-a-year business [37, 38]. Since RMLs have been expected to increase availability, advertising, and accepting attitudes towards cannabis use, an increase in people who are users and thus increases in population rates of adverse health or psychosocial consequences of cannabis use has been expected as well.

## Effects of Marijuana Laws: Trends in Cannabis Potency

The primary psychoactive component in cannabis is delta-9-tetrahydrocannabinol (THC). THC directly targets the body’s natural endogenous endocannabinoid system, including the receptors that mediate the direct actions of cannabinoids [23]. Cannabinoid CB_1_ receptors, which mediate the action of THC, are particularly concentrated in brain regions such as the hippocampus and amygdala, basal ganglia, anterior cingulate cortex, and prefrontal cortex [39]. These brain regions are associated with memory, attention, psychomotor (related to driving), inhibitory control, and higher executive functions. Cannabis potency is generally defined as the percent of THC per volume amount of the marijuana product.

In samples of illegal cannabis seized by law enforcement between 1990 and 2010 [40], mean THC potency was higher in states that passed MML (9.1%) than in other states (5.6%). Potency of cannabis products has increased since then. In Washington (where RML was first legalized in 2012), the average THC potency of marijuana for one Seattle-based retailer in 2015 was 21.2% [41]. Colorado also first passed RML in 2012. There, the THC potency of legally marketed cannabis can range considerably, with some strains having potencies of 28–32% [42]. Further, while smoking remains the most common route of administration, other routes of administration are increasingly common [43–45], including edibles, vaping (inhaled vapor of heated e-liquids analogous to e-cigarettes), and dabbing (inhaled vapor from heating highly-concentrated forms of cannabis or hashish). These routes offer higher THC doses than typically smoked plant marijuana in joints [43]. A recent study of online cannabis advertising showed that the mean potency of medical and recreational marijuana products was similar, 19.2 and 21.5%, respectively [46]. Because cannabis potency is related to effects on cognition, the generally stronger potency of available cannabis products may be increasingly harmful in both acute and chronic use and persist longer in abstinence. Future studies on this are needed. In the meantime, given the overall increases in potency since the 1990s accompanying changing laws and attitudes, recent neurocognitive or neuroimaging studies that include people who are cannabis users may be more impacted by cannabis than studies conducted many years ago, which should be taken into account in interpreting the results of studies done during different periods, e.g., during the 1990s vs very recent years. This issue clearly applies to groups or conditions where people who are cannabis users are the main group of interest. However, studies with other groups or conditions of primary interest may also be impacted by the increases in potency if participants are not carefully tested for recent cannabis use and excluded if they show signs of recent use.

## Effects of Marijuana Laws: Trends in Adolescents Who Use Cannabis

Concerns that MML would increase adolescent cannabis use emerged about 10 years ago [47, 48], based on the possibility that MML would conveying a message that marijuana is acceptable or lacks negative consequences [49•]. An early study of national data showed that adolescent cannabis use was associated with residing in states with MML, which appeared to confirm the fears about MML effects on teens [50]. However, as Wall et al. stated in the paper, cross-sectional associations do not indicate causality. Therefore, subsequent studies of national data used difference-in-difference (DiD) methods to examine changes in state rates before and after MML passage compared with contemporaneous changes in states that did not change their marijuana laws [51–54]. Of 17 large surveys using DiD methods spanning different states, periods, and specifications, 16 indicated no MML effects on adolescent use [28, 31, 47, 55–60]. Thus, despite methodological differences between the studies, their findings were very consistent: rates of adolescent cannabis use did not increase post-MML compared with pre-MML or to national trends in non-MML states during the corresponding years.

To our knowledge, only one study to date used national data to examine the effects of recreational marijuana laws (RML) on adolescent marijuana use that analyzed data appropriate for this purpose [61•]. This study found no effects of RML on adolescent past-year use or frequent use and a weak effect on the risk of cannabis use disorder that was not robust in sensitivity analyses. A different study suggested decreases in adolescent use post-RML [62]. However, the methods of this study have been disputed [63–66]. Taken as a whole, the current literature does not indicate that increasingly permissive state marijuana laws, either medical marijuana or recreational laws, increase the prevalence of marijuana use in adolescents. Therefore, these laws appear not to have current implications for behavioral neuroscience studies of adolescents. However, continued studies and monitoring of the literature on cannabis laws and adolescents is important to determine if MML or RML effects on teen cannabis use begin to emerge.

## Effects of Marijuana Laws: Adult Cannabis Use and Cannabis Use Disorders

**Fig. 2:**
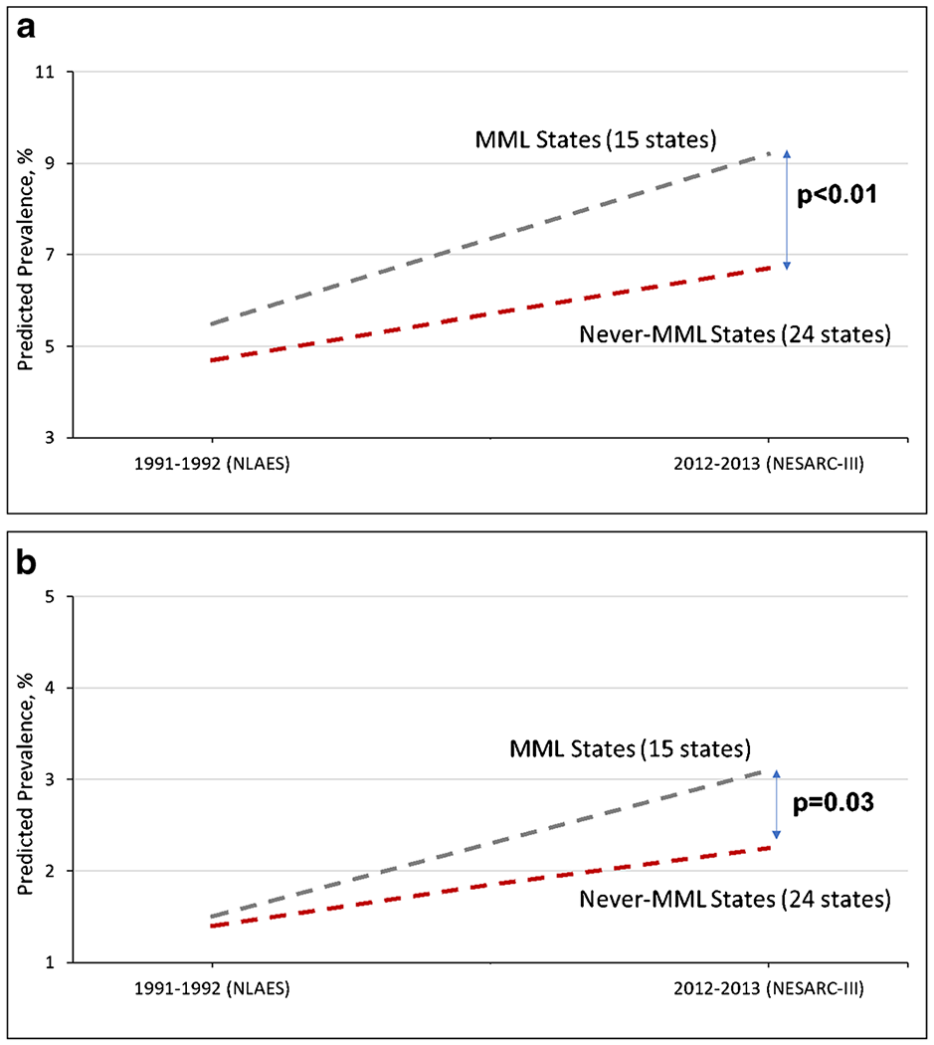
**a** Non-medical cannabis use in US adults, 1991–1992 to 2012–2013, by MML state status Abbreviations: MML – Medical Marijuana Law (adapted from Hasin et al., JAMA Psychiatry 2017). **b** DSM-IV Cannabis use disorder in US adults, 1991–1992 to 2012–2013, by MML state status

In contrast to the relatively large literature on adolescents and MML effects, fewer studies investigated the relationship of MML or RML to adult cannabis use or related outcomes. A cross-sectional analysis of national 2004–2005 data showed higher rates of adult cannabis use and cannabis disorders in MML than in non-MML states [67]. However, studies using DiD tests were needed to examine causality. Indirectly, a study suggested MML effects on adult cannabis use by showing a 15–20% post-MML increase in urban adult marijuana possession arrests [68] and a 20% post-MML increase in first-time adult marijuana treatment admissions [68]. Using 2004–2013 data from the National Survey on Drag Use and Health (NSDUH) at a point when 10 states had passed MMLs, DiD tests indicated significant post-MML increases for adult cannabis use, daily or near-daily use, and 1- and 2-year lagged effects on CUD [31]. For cannabis use, this effect was confirmed in adults age 26 and older [56]. Using three national survey datasets from 1991 to 1992 to 2012–2013 to compare 15 MML states to other states, post-MML increases were found for adult cannabis use (Fig. 2a and CUD (Fig. 2b) [69•]. Using the same three datasets (1991–1992 to 2012–2013), post-MML increases were found in driving under the influence of cannabis [70] but not alcohol, indicating that the MML effects were substance-specific.

A study of RML effects on adults in yearly national surveys from 2008 to 2016 found no effects in young adults age 18–25 [61•]. However, among adults age 26 and older, post-RML increases were found for past-year cannabis use, frequent use, and cannabis use disorders. Thus, while the adult research base is not extensive, existing studies are consistent in showing post-MML and post-RML increases in adult cannabis-related outcomes.

The findings on the post-MML and -RML increases in cannabis use and cannabis use disorders indicate that increasingly permissive state marijuana laws do have implications for behavioral neuroscience studies of adults, particularly those age 26 and older. Potential participants in studies conducted in states with MML and particularly RML are more likely to be people who are cannabis users and frequent users than participants in other states. For studies comparing people who are cannabis users or those with cannabis use disorders to cannabis-naïve controls, this may only affect feasibility since the pool of cannabis-naïve participants that can potentially be recruited will be smaller. However, the scientific findings of studies focused on other groups or conditions of interest may be impacted in a more serious way if participants are not carefully tested for recent cannabis use and excluded if they test positive. This may particularly affect studies of neurocognitive or brain functioning studies of depression or anxiety disorders. Cannabis withdrawal syndrome as defined in the *Diagnostic and Statistical Manual of Mental Disorders, Fifth Edition (DSM-5)* consists of at least 3 of the following symptoms developing within 7 days of reduced cannabis use: (1) irritability, anger, or aggression; (2) nervousness or anxiety; (3) sleep disturbance; (4) appetite or weight disturbance; (5) restlessness; (6) depressed mood; and (7) somatic symptoms, such as headaches, sweating, nausea, vomiting, or abdominal pain. The duration of this syndrome post-abstinence is unclear, but about half of people who are regular cannabis users experience cannabis withdrawal syndrome [71]. Because many cannabis withdrawal symptoms overlap with symptoms of depressive or anxiety disorders, many people who are regular cannabis users may continue using cannabis in an effort at self-medication of these depression or anxiety symptoms, unaware that this use could perpetuate a longer-term withdrawal problem [49]. In such users, if the cannabis use history is not known, cannabis withdrawal could easily be confused with depressive or anxiety disorders by clinicians or research investigators, leading to results and inferences that are unclear or potentially incorrect.

## People Who Are Medical Cannabis Users Vs. People Who Are Recreational Cannabis Users

Studies of the relationship of marijuana to neurocognition and neurofunctioning have largely focused on samples of people who are recreational marijuana users. However, over two-thirds of US states now permit legal use of marijuana for medical purposes, raising issues about potential implications of medical marijuana use for neuroscience research. These include questions about whether people who are medical users are different from people who are recreational users, and whether cannabis may differentially affect people who are medical users. Several early studies from California, the first state to pass MML, showed that people who were medical and recreational users had similar characteristics and that people who were medical users often had histories of recreational use [72–76]. These studies suggested that in these early days of medical marijuana, the medical authorizations were often obtained by people who were recreational users. However, recent reports on medical marijuana users from national surveys provide more current, representative information. In data from the National Survey on Drag Use and Health (NSDUH), among participants who were cannabis users from MML states, those who were medical users were less likely than those who were recreational users to have good health or substance use disorders (Lin, et al. 2016). In another NSDUH study of all states, those who were medical cannabis users had poorer health, worse rdisability, older age, and late initiation of cannabis use than others [77].

Behaviors such as use and misuse of illicit substances often cluster empirically within the externalizing domain of psychopathology [78–81], typically with early onset, common etiology, and traits including sensation-seeking and impulsivity [82–84]. People who are recreational cannabis users seeking the sensation of feeling high are likely to differ from people who are medical patients [85, 86] seeking marijuana for relief of pain or other symptoms and who are not typically characterized by externalizing traits [87, 88]. People who are medical marijuana users may therefore differ in numerous ways from people who are recreational users in whom cognitive effects have mainly been studied to date, including later onset of use and complicating medical problems. They may also have a different set of heritable personality traits that will be reflected in results of neurocognitive and imaging studies. Studies of cannabis and cognition in people who are medical marijuana users are needed [85, 86] to determine whether marijuana effects on cognition and brain functioning differ in such users from the people who are recreational users who have been studied more extensively. In the end, such studies may provide a broader, richer understanding of the relationship of cannabis to neurofunctioning.

## Conclusions

In summary and conclusion, the population prevalence of people who are regular or heavy use of cannabis among US adults has increased over the last 20 years, medical marijuana use is now largely permitted across the USA, and adult residents of states that have legalized cannabis are more likely to be people who use cannabis recreationally and to have cannabis use disorders. Therefore, when evaluating participant eligibility for studies of neurocognitive or brain functioning, carefully evaluating a history of recent use (recreational or medical) via interviewing and biological tests will be increasingly important to avoid misleading results or simply reduce error variance in such studies. Additionally, for clinical care, careful histories about marijuana use at the beginning of treatment could avoid diagnostic confusion and provide a better basis for treatment planning, monitored by periodic check-ins on marijuana use as treatment proceeds.
